# The Basolateral Amygdalae and Frontotemporal Network Functions for Threat Perception

**DOI:** 10.1523/ENEURO.0314-16.2016

**Published:** 2017-03-27

**Authors:** Ruud Hortensius, David Terburg, Barak Morgan, Dan J. Stein, Jack van Honk, Beatrice de Gelder

**Affiliations:** 1 Brain and Emotion Laboratory, Department of Cognitive Neuroscience, Faculty of Psychology and Neuroscience, Maastricht University, Oxfordlaan 55, 6229 EV Maastricht, The Netherlands; 2 Department of Psychiatry and Mental Health, University of Cape Town, J-Block, Groote Schuur Hospital, Observatory, Cape Town, South Africa; 3 Experimental Psychology, Utrecht University, Heidelberglaan 1, 3584 CS Utrecht, The Netherlands; 4 Global Risk Governance Programme, Institute for Safety Governance and Criminology, Law Faculty, University of Cape Town, University Avenue, Rondebosch 7700, Cape Town, South Africa; 5 DST-NRF Centre of Excellence in Human Development, DVC Research Office, University of Witwatersrand, York Road, Parktown, Johannesburg, South Africa; 6 Department of Psychiatry and Medical Research Council (MRC) Unit on Anxiety & Stress Disorders, University of Cape Town, J-Block, Groote Schuur Hospital, Observatory, Cape Town, South Africa; 7 Institute of Infectious Diseases and Molecular Medicine (IDM), University of Cape Town, Anzio Road, Observatory 7925, Cape Town, South Africa; 8 Department of Computer Science, University College London, Gower Street, London WC1E 6BT, United Kingdom

**Keywords:** amygdalae, basolateral amygdalae, emotion, threat, Urbach–Wiethe disease

## Abstract

Although the amygdalae play a central role in threat perception and reactions, the direct contributions of the amygdalae to specific aspects of threat perception, from ambiguity resolution to reflexive or deliberate action, remain ill understood in humans. Animal studies show that a detailed understanding requires a focus on the different subnuclei, which is not yet achieved in human research. Given the limits of human imaging methods, the crucial contribution needs to come from individuals with exclusive and selective amygdalae lesions. The current study investigated the role of the basolateral amygdalae and their connection with associated frontal and temporal networks in the automatic perception of threat. Functional activation and connectivity of five individuals with Urbach–Wiethe disease with focal basolateral amygdalae damage and 12 matched controls were measured with functional MRI while they attended to the facial expression of a threatening face–body compound stimuli. Basolateral amygdalae damage was associated with decreased activation in the temporal pole but increased activity in the ventral and dorsal medial prefrontal and medial orbitofrontal cortex. This dissociation between the prefrontal and temporal networks was also present in the connectivity maps. Our results contribute to a dynamic, multirole, subnuclei-based perspective on the involvement of the amygdalae in fear perception. Damage to the basolateral amygdalae decreases activity in the temporal network while increasing activity in the frontal network, thereby potentially triggering a switch from resolving ambiguity to dysfunctional threat signaling and regulation, resulting in hypersensitivity to threat.

## Significance Statement

Humans are experts in recognizing potential threat signals. Although the role of the human amygdalae is widely acknowledged, the contributions of the different amygdalae nuclei and associated neural networks in threat perception remain poorly understood. Here we investigate the importance of the basolateral amygdalae and their connections with temporal and frontal regions during the processing of task-irrelevant threatening bodily signals. We tested five individuals who have selective basolateral amygdalae damage. The results show that after basolateral amygdalae damage, activity was increased in the frontal network but decreased in the temporal network. Together with anomalous activity in regions important for action, these results point to a disruption along three axes during threat perception, namely ambiguity resolution, safety signaling, and action preparation.

## Introduction

It is widely acknowledged that the amygdalae (AMG) play a central role in threat processing. Neuroimaging studies in healthy individuals have shown that the AMG are activated in response to seeing facial expressions (Morris et al., 1996; Sabatinelli et al., 2011) as well as bodily expressions of threat (Hadjikhani and de Gelder, 2003; de Gelder et al., 2012). However, in humans our understanding remains patchy, and the specific contribution to different aspects of threat perception, from ambiguity resolution to safety signaling and action, cannot yet be disentangled. For a better understanding of the central role of the AMG in threat perception, it is essential to distinguish the role of the different nuclei and map their specific connectivity profile (Hortensius et al., 2016a). Given the limitations of human imaging methods, the contribution of lesion studies is crucial (Adolphs, 2016; Madarasz et al., 2016).

The major division of the AMG is between the superficial (SFA), basolateral (BLA), and central-medial (CMA) amygdalae (McDonald, 1998). This subdivision corresponds to three different networks, the olfactory network (SFA), the autonomic network (CMA), and the frontal-temporal network (BLA; Swanson and Petrovich, 1998; Bzdok et al., 2013). The latter two networks are specifically important for threat processing and behavior. The CMA mediate reflexive reactions to threat together with the hypothalamus and brainstem (Mosher et al., 2010; Fox et al., 2015). The role of the BLA in threat perception and action is more complex. The BLA receive input from the sensory thalamus and sensory cortices and have bidirectional connections with many cortical, including frontal and temporal, regions such as the ventral and dorsal part of medial prefrontal cortex (MPFC) and temporal pole (TP; Heimer et al., 1997; Ghashghaei and Barbas, 2002). The BLA-temporal network plays a role in the emotional labeling of ambiguous object categories and affective value calculation (Benarroch, 2015). The connections with the medial and orbital part of the prefrontal cortex underlies safety signaling, emotion regulation, and affective learning (Likhtik and Paz, 2015). The BLA are crucial in the perception and reaction to facial and bodily expressions and are particularly sensitive to ambiguity (Madarasz et al., 2016); this might especially be the case during a possible mismatch between these expressions.

Information from the face and the body is sampled and combined at an early stage, around 115 ms after stimulus onset (Meeren et al., 2005). Bodily expressions influence recognition of facial expressions (Meeren et al., 2005; Van den Stock et al., 2007; Aviezer et al., 2008, 2012a, 2012b), face identity recognition (Van den Stock and de Gelder, 2014) and memory (Van den Stock and de Gelder, 2012). For instance, the interpretation of a happy face combined with an angry body can be biased toward the latter (Kret and de Gelder, 2013). Recent behavioral evidence showed a crucial role of the BLA in the integration of face–body information. Three individuals with bilateral BLA damage showed a deficit in ignoring task-irrelevant threatening bodily expressions during emotion face recognition (de Gelder et al., 2014). The question remains how the BLA together with the temporal and frontal networks process task-irrelevant bodily threat signals and how activity in these networks changes after BLA damage.

In the present functional MRI (fMRI) study, we investigated the neural basis of perceiving threatening facial and bodily expressions in isolation or in congruent (matching) or incongruent (mismatching) face–body compounds in five participants with specific BLA calcification and 12 matched controls. The goal of our study was to clarify the effect of BLA damage on activity in the frontal and temporal networks during irrelevant threat processing. The previously reported behavioral finding of excessive influence of task-irrelevant and unattended bodily expressions on facial expression recognition after BLA damage could be the result of disruption in the BLA-frontal or the BLA-temporal network and point to a mechanism rooted in either threat signaling or emotion integration and interpretation, respectively, or a combination. The BLA, by activating inhibitory neurons in the MPFC, have an inhibitory influence on the MPFC (Dilgen et al., 2013), and damage to the BLA might result in an increase in activation in both the dorsal and ventral part of the MPFC. In contrast, it has been reported that long-term damage to the entire AMG resulted in structural changes in visual and temporal regions (Boes et al., 2012). BLA damage will most likely also disrupt activity in the BLA-temporal network, but the exact functional consequences are at present unknown (Vuilleumier et al., 2004; Edmiston et al., 2013).

## Materials and Methods

### Participants

**Figure 1.**
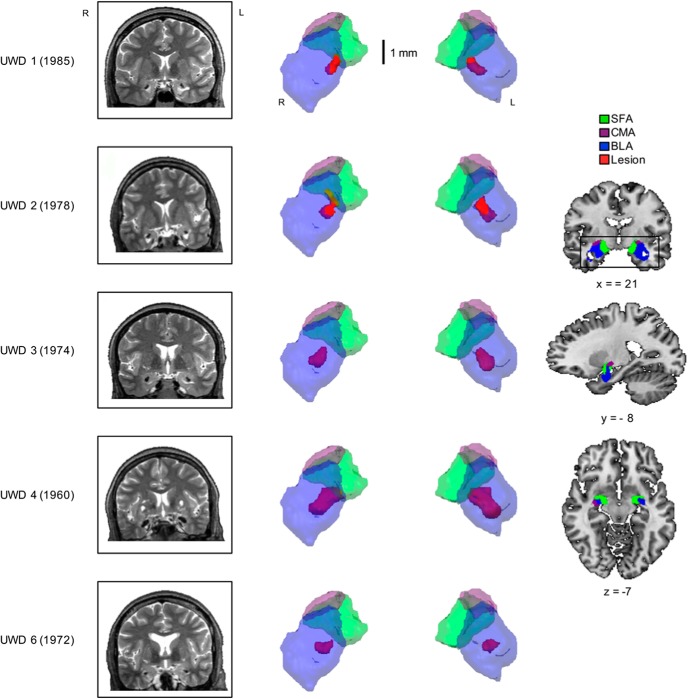
Location and size of the BLA damage. Coronal view of T2-weighted MRIs (left) and a 3D reconstruction (middle) of the lesion for the five individuals with UWD with birth year indicated. Reconstruction of the AMG subnuclei was based on the cytoarchitectonic probability maps from Amunts et al., (2005) in Eickhoff et al., (2005). Black rectangle indicates viewpoint for the 3D reconstruction (right).

Five volunteers with Urbach–Wiethe disease (UWD) from the Northern Cape of South Africa (Thornton et al., 2008) and 12 matched controls from the same region participated in the present experiment (all women). Participants had no history of secondary psychopathology or epileptic insults. Environmental conditions, age, and neuropsychological characteristics were similar for the UWD and control group (Table 1). UWD is a disease that in some cases includes bilateral calcification of the AMG. See Fig. 1 and Movie 1 for the location and size of the calcification and 3D reconstruction of the lesion. Previously, structural MRI and fMRI assessment by means of cytoarchitectonic-probability labeling provided evidence that the calcification is restricted to the BLA (Terburg et al., 2012; Klumpers et al., 2015b). Three of the five individuals with UWD (UWD 1–3) also participated in the previously reported behavioral experiment (de Gelder et al., 2014) using a design similar to the one used in the present study. The three individuals with UWD showed a large and significant deficit in ignoring task-irrelevant bodily threat compared with controls (effect size, *r* ≥ –0.58). Participants were unaware of the aim of the study and provided written informed consent. The study was approved by the Health Sciences Faculty Human Research Ethics Committee of the University of Cape Town and conducted in accordance with the standards set by the Declaration of Helsinki.

**Table 1.** Demographic data

	UWD (*n* = 5)						Controls (*n* = 12)
Characteristic	UWD 1	UWD 2	UWD 3	UWD 4	UWD 6	Mean	Mean
Age	27	34	38	52	39	38 ± 9.14	37.17 ± 5.20
VIQ	97	84	93	82	83	87.80 ± 6.76	86.67 ± 4.68
PIQ	99	87	85	84	87	88.40 ± 6.07	88.17 ± 5.39
FSIQ	98	84	87	81	83	86.60 ± 6.73	85.83 ± 4.43

VIQ, verbal IQ; PIQ, performance IQ; FSIQ, full-scale IQ. Means and SDs are reported. No significant differences between groups, *p* ≥ 0.78.

**Movie 1.** Three-dimensional reconstruction of the BLA lesion for the five individuals with UWD with birth year indicated.

### Stimuli and task

Compound stimuli were created by combining facial and bodily expressions (Meeren et al., 2005). Fearful and happy faces (MacBrain Face Stimulus Set) were paired with a fearful or happy body (de Gelder and Van den Stock, 2011), resulting in congruent (e.g., a fearful face with a fearful body) or incongruent (e.g., a happy face with a fearful body) compounds. To create compound stimuli showing only facial or bodily expressions, the face or body was replaced with a gray shape (e.g., a happy face with gray rectangle, a gray oval with a fearful body). An additional control compound stimulus was created in which both facial and bodily expressions were replaced by a gray oval and gray rectangle. We used gray shapes instead of neutral expressions, as neutral expressions are often not perceived as neutral and are evaluated on multiple dimensions (Todorov et al., 2008), for example dominance (Mignault and Chaudhuri, 2003; Oosterhof and Todorov, 2008) and emotion (Malatesta et al., 1987; Said et al., 2009), and the processing of these faces is influenced by the rest of the body (Van den Stock and de Gelder, 2012, 2014). Ten unique stimuli (five female) per condition were created.

Participants performed a passive oddball task (Carretié et al., 2004). In this task, participants focused on the fixation cross placed on the nose of the face. Thus, attention of the participants was on the face and not on the rest of the body. During the task, an oddball stimulus could appear that would have a red circle overlaid on the nose of the face instead of a black fixation cross. Participants were instructed to pay attention to this change, but did not have to make an overt response. This was done to counteract any possible contamination of the blood-oxygenation-level dependent signal (BOLD) by a motor response. A nurse familiar to the participants was trained to provide instructions outside of the scanner. The task was explained to the participant with examples of face–body compound stimuli not used in the actual experiment. The experiment started when participants indicated that they understood the instructions.

A block design was used. During a stimulation block, the 10 stimuli belonging to the same category (e.g., fearful face with a happy body) were presented in a random order for 800 ms each, with an interstimulus interval of 200 ms (total duration 10 s). Each run consisted of 27 stimulation blocks (nine different conditions repeated three times) and six oddball blocks presented in a random order. This was followed by an interblock interval of 6 s. Three rest blocks of 10 s each were presented at a fixed time point (after stimulation/oddball blocks 5, 11, and 22). To counteract any possible habituation and provide a more dynamic presentation, no stimuli were shown during these rest blocks. Participants completed two runs, lasting 18 min in total. Stimuli were presented using E-Prime 2.0 software (Psychology Software Tools), projected onto a screen located at the end of the scanner bore. Each new event was synchronous with a new scan volume.

### Image acquisition

Data were acquired with a Siemens Magnetom Allegra 3 Tesla head-only scanner (Siemens Medical Systems) at the Cape Universities Brain Imaging Center in Cape Town, South Africa. Participants were fitted with earplugs to attenuate the scanner noise, and padding was used to reduce head movements. Functional whole brain coverage was achieved using 2D echo-planar images sequence. Each volume contained 36 slices acquired in ascending order with a 3.5-mm isotropic resolution (interslice gap = 0.525, TR = 2000 ms, TE = 27 ms, flip angle = 70°, field of view = 225 × 225 mm^2^, matrix size = 64 × 64). In total, 278 functional volumes were collected per run. After the final functional run, a high-resolution T1-weighted anatomic scan with 1-mm isotropic resolution was collected (no gap, TR = 2300 ms, TE = 39 ms, FA = 9°, field of view = 240 × 256 mm^2^, matrix size = 256 × 256).

### fMRI preprocessing and analyses

Data preprocessing and analyses were conducted using BrainVoyager QX Version 2.8.4 (Brain Innovation). The first four volumes of each run were discarded from analyses to avoid T1 saturation effects. Preprocessing of the functional data consisted of slice time correction (using sinc interpolation), a rigid-body algorithm to correct for small movements between scans (trilinear/sinc estimation and interpolation), and temporal high-pass filtering (GLM-Fourier with two cycles sine/cosine per run including linear trend removal). No spatial smoothing was used. Functional data were coregistered to the anatomic data, and all data were normalized into Talairach space.

To reduce individual macro-anatomic differences between participants and crucially between the UWD and control group, and to subsequently improve statistical power, cortex-based alignment (CBA) was used (Goebel et al., 2006; Frost and Goebel, 2012). This high-resolution cortical mapping procedure achieves a nonrigid alignment of different brains using the individual curvature information that reflects the gyri and sulci folding patterns (Frost and Goebel, 2012). Because the CBA procedure already applies smoothing to the data and results in superior alignment between participants, no further spatial smoothing was used.

At the single-subject level, a fixed-effects whole-brain general linear model was applied with each condition and oddball block defined as predictors. The z-transformed motion predictors were included as predictors of no interest. In addition, to reduce error variance, outlier predictors were included in the model (Luo and Nichols, 2003; Carter et al., 2008). An outlier map was created for each run of each participant to show clusters that had a time course value of >6 SD above the mean. The clusters in these maps were manually inspected, and if the value was >6 SD above the mean, but not related to motion or an incidental spike, the time course was extracted, z-transformed, and included in the design matrix. Next, the design matrix of each run of each participant was checked and corrected for shared variance. Predictors of no interest explained by a combination of other predictors (*R* ^2^ > 0.80) were removed from the design matrix. For example, if *Y* rotation estimates were explained by the other (motion) predictors, *Y* rotation estimates were not included in the model. Thus, besides the task predictors (nine + one oddball), motion predictors and possible outlier predictors were included in the design matrix. The number of predictors of no interest did not differ between groups (*p* > 0.22) and ranged between five and nine across subjects.

At the group level, a random-effects general linear model was applied. Using a dummy-coded general linear model, the following main analyses were performed. First, we investigated the regions that were activated more for fearful compared with happy bodies regardless of the facial information. Second, to map the effect of incongruent versus congruent face–body compounds, we contrasted incongruent (fearful face and happy body or happy face and fearful body) with congruent (fearful face and fearful body or happy face and happy body). Third, to determine the influence of task-irrelevant fear versus task-irrelevant happiness, fearful bodies with a happy face or gray oval were contrasted with happy bodies with fearful face or gray oval.

Between-group as well as within-group maps (for the UWD and control group separately as well as combined) were calculated. The between-group maps were cluster size corrected (Forman et al., 1995). In brief, a whole-brain correction was calculated by estimating a false-positive rate for each cluster by taking into account the spatial smoothness of the initial statistical map. In accordance with Goebel et al. (2006), the initial single voxel threshold was set at *p* = 0.01, and the minimal cluster size threshold applied to the final statistical maps after Monte Carlo simulation (1000 iterations) corresponded to a cluster-level false-positive rate (α) of 5%. Whereas it has been argued that an initial threshold of *p* = 0.001 is recommended (Woo et al., 2014), we chose a more liberal threshold given the special population and methodological steps (CBA, random-effects general linear model, no spatial smoothing). A more lenient threshold is advised to avoid type II errors and counteract activation pattern biases (large versus small effects and dominance of visual regions; Lieberman and Cunningham, 2009). The individual and combined group maps of the UWD and control groups were tested against zero using a one-sample *t* test and thresholded at *p* < 0.01, with an extended cluster size of 25.

Next, besides testing for differences in functional segregation, we established differential functional integration by performing connectivity analyses (Price et al., 2006). We used psychophysiological interaction (Friston et al., 1997) to probe the potential impact of BLA damage on the neural network underlying threat perception. Functional coupling between the seed region identified in the between-group analyses and other regions was estimated as a function of the psychological context. The demeaned extracted time course from the seed region (the physiological state) was used to create psychophysiological interaction predictors by multiplying it with the contrast of interest (psychological state). Besides psychophysiological interaction and contrast predictors, the time course of the seed region, motion, and possible outlier predictors were included in the model. After the fixed-effects single-subject analysis, a whole-brain random effects group analysis was used to map the difference in connectivity pattern between the UWD and control group. Thresholds were similar as in the functional activation analyses. All statistical maps are shown on the average group-aligned surface reconstruction and Talairach coordinates, and *t*- and *p*-values of peak vertices are reported.

## Results

### Functional activation

No between-group differences were found when contrasting emotional faces or bodies versus control stimuli. Tables 2 and 3 report the significant clusters for the UWD and control groups combined. No significant clusters were found between or within groups for fearful versus happy facial expression regardless of bodily expression. These functional maps are in line with previous research on face and body perception (van de Riet et al., 2009; de Gelder et al., 2010; Kret et al., 2011; Sabatinelli et al., 2011). Moreover, the lack of significant differences in functional activation between individuals with UWD and controls when perceiving emotional faces and bodies in isolation is in line with behavioral observations of intact emotion recognition of both facial and bodily expressions in isolation (Terburg et al., 2012; de Gelder et al., 2014).

**Table 2.** Fearful and happy faces > control stimuli for both the UWD and control group

		Talairach coordinates						
	Hemisphere	*x*	*y*	*z*	Brodmann	*t*	*p*	Number of vertices
Inferior occipital gyrus	RH	27	–87	–9	18	6.756	0.000005	183
Fusiform gyrus	RH	35	–55	–13	37	6.321	0.00001	133
Lingual gyrus	RH	8	–72	4	18	5.199	0.000088	39
Inferior occipital gyrus	LH	–29	–84	–7	18	8.947	<0.000001	717
Middle frontal gyrus	LH	–18	18	53	6	4.924	0.000153	42
Cuneus	LH	–7	–81	4	17	4.983	0.000136	90
Precuneus	LH	–20	–63	49	7	4.411	0.000437	76
Superior frontal gyrus	LH	–20	45	31	9	6.493	0.000007	109

*p* < 0.01 (uncorrected) with an extended cluster size of 25. Faces are presented with a gray rectangle, and the control stimulus is a gray oval and rectangle.

**Table 3.** Fearful and happy bodies > control stimuli for both the UWD and control group

		Talairach coordinates						
	Hemisphere	*x*	*y*	*z*	Brodmann	*t*	*p*	Number of vertices
Lingual gyrus	RH	15	–84	–11	18	5.323	0.000069	93
Fusiform gyrus	RH	41	–59	–13	37	7.222	0.000002	147
Middle occipital gyrus	RH	28	–89	2	18	4.631	0.000277	32
Inferior occipital gyrus	RH	27	–87	–9	18	5.547	0.000044	47
Cuneus	RH	8	–90	11	18	4.235	0.000631	18
Middle occipital gyrus	RH	36	–76	9	19	4.616	0.000286	39
Inferior occipital gyrus	LH	–12	–90	–10	17	8.011	0.000001	1189
Precuneus	LH	–20	–58	55	7	4.441	0.000411	122
Superior frontal gyrus	LH	–6	51	29	9	6.979	0.000003	151
Precuneus	LH	–24	–71	21	31	4.608	0.000291	74
Parahippocampal gyrus	LH	–21	–52	5	30	4.706	0.000238	48
Superior frontal gyrus	LH	–20	10	55	6	4.732	0.000226	37
Posterior cingulate	LH	–6	–50	19	30	4.238	0.000627	56
Precentral gyrus	LH	–29	–9	48	6	4.762	0.000212	90
Superior frontal gyrus	LH	–9	62	16	10	4.774	0.000207	18

*p* < 0.01 (uncorrected) with an extended cluster size of 25. Bodies are presented with a gray oval, and the control stimulus is a gray oval and rectangle.

To add to behavioral and electroencephalograph studies on face-body compound perception (Meeren et al., 2005; Kret and de Gelder, 2013; de Gelder et al., 2014) and establish the functional activation in the presence of functional BLA, we report the functional maps in the control group separately (Tables 4–6). Results revealed no regions that were activated more for fearful compared with happy bodies regardless of the facial information. Second, the temporal pole [TP; Brodmann area (BA) 21], superior (BA 38), and inferior temporal gyrus (BA 20) were activated for happy versus fearful bodies regardless of the facial information. Third, significant clusters were observed for congruent (a fearful face with a fearful body or a happy face with a happy body) versus incongruent face–body compounds (a fearful face with a happy body or a happy face with a fearful body), but not for the inverse contrast. Activity increased for congruent compared with incongruent compounds in the superior frontal gyrus (BA 6) and ventromedial prefrontal cortex (BA 10). Last, we tested the specific effect of task-irrelevant fearful versus happy bodies, that is fearful bodies combined with a happy face or a gray oval versus happy bodies with fearful faces or a gray oval. For this contrast, the cingulate gyrus (BA 23) and cuneus (BA 18) were activated for task-irrelevant fear bodies compared with task-irrelevant happy bodies.

**Table 4.** Fearful versus happy bodies regardless of the facial information

		Talairach coordinates						
	Hemisphere	*x*	*y*	*z*	Brodmann	*t*	*p*	Number of vertices
Controls								
Happy > fear								
Temporal pole	RH	38	–3	–30	21	–3.636	0.002225	42
Superior temporal gyrus	RH	49	9	–9	38	–2.919	0.010028	39
Inferior temporal gyrus	LH	–50	–16	–25	20	–3.182	0.005790	30
UWD								
No significant clusters								
UWD and controls								
No significant clusters								

*p* < 0.01 (uncorrected) with an extended cluster size of 25.

**Table 5.** Incongruent versus congruent face-body compounds

		Talairach coordinates						
	Hemisphere	*x*	*y*	*z*	Brodmann	*t*	*p*	Number of vertices
Controls								
Congruent > incongruent								
Superior frontal gyrus	RH	9	26	54	6	–3.996	0.001040	30
Ventromedial prefrontal cortex	RH	8	41	–1	10	–2.414	0.028110	34
UWD								
Congruent > incongruent								
Insula	RH	36	–8	6	13	–3.093	0.006981	46
Insula	LH	–34	–4	3	13	–2.608	0.019014	51
UWD and controls								
Congruent > incongruent								
Inferior parietal lobule	LH	–32	–46	37	40	–5.817	0.000026	68

*p* < 0.01 (uncorrected) with an extended cluster size of 25.

**Table 6.** Task-irrelevant fear versus task-irrelevant happiness

		Talairach coordinates						
	Hemisphere	*x*	*y*	*z*	Brodmann	*t*	*p*	Number of vertices
Controls								
Task-irrelevant fear > task-irrelevant happiness								
Cingulate gyrus	RH	2	–12	27	23	6.603	0.000006	47
Cuneus	LH	–3	–71	13	18	2.964	0.009131	25
UWD								
Task-irrelevant fear > task-irrelevant happiness								
Cingulate gyrus	RH	4	–10	37	24	6.741	0.000005	58
Task-irrelevant happiness > task-irrelevant fear								
Middle frontal gyrus	LH	–41	16	26	46	–3.000	0.008479	33
UWD and controls								
Task-irrelevant fear > task-irrelevant happiness								
Cingulate gyrus	RH	4	–10	37	24	6.741	0.000005	50
Cingulate gyrus	RH	2	–12	27	23	6.603	0.000006	51

*p* < 0.01 (uncorrected) with an extended cluster size of 25.

**Figure 2.**
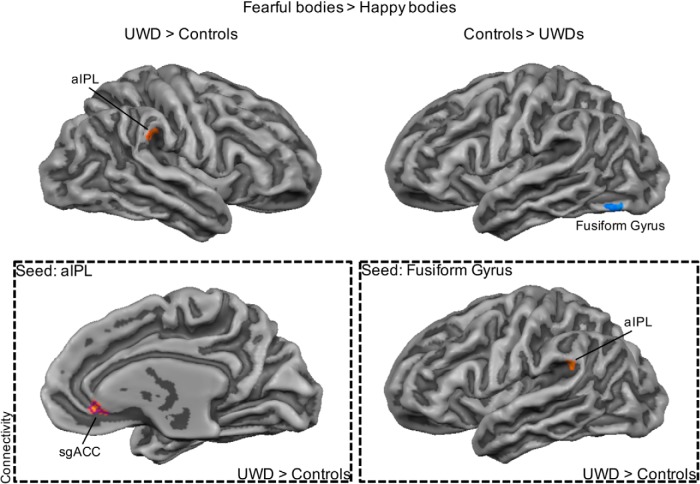
The importance of the IPL in the processing of fearful body expressions. The UWD group showed more activation for fearful versus happy bodies in the right anterior IPL, but less activation in the left fusiform gyrus (top). Increased functional connectivity between the IPL and the subgenual ACC, and the fusiform gyrus and the anterior IPL, was observed in individuals with UWD compared with controls (bottom). Purple outline indicates that the cluster survived whole-brain cluster-size correction with an initial single voxel threshold of *p* < 0.005.

**Figure 3.**
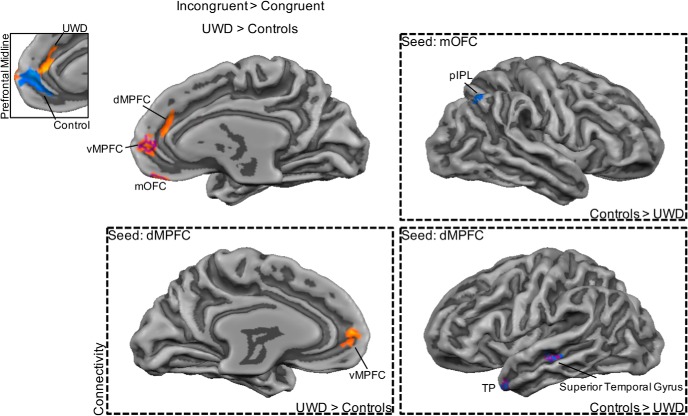
Enhancement of prefrontal midline activation during the perception of incongruent threatening face–body compounds after BLA damage. The mOFC, vMPFC, and dMPFC showed increased activity in the UWD group (top left) during incongruent threatening face–body compound perception. Inset shows increased dMPFC activation for incongruent versus congruent face–body compounds in individuals with UWD and decreased vMPFC activation for the same contrast in controls. Individuals with UWD showed decreased functional connectivity between the mOFC and the posterior IPL. The dMPFC showed increased coupling with the VMPFC, but decreased coupling with the superior temporal gyrus and TP in individuals with UWD (right and bottom). Maps are cluster-size corrected except for the within-group maps that are shown with a threshold of *p* < 0.05 uncorrected for illustration purposes. Purple outline indicates that the cluster survived whole-brain cluster-size correction with an initial single voxel threshold of *p* < 0.005.

**Figure 4.**
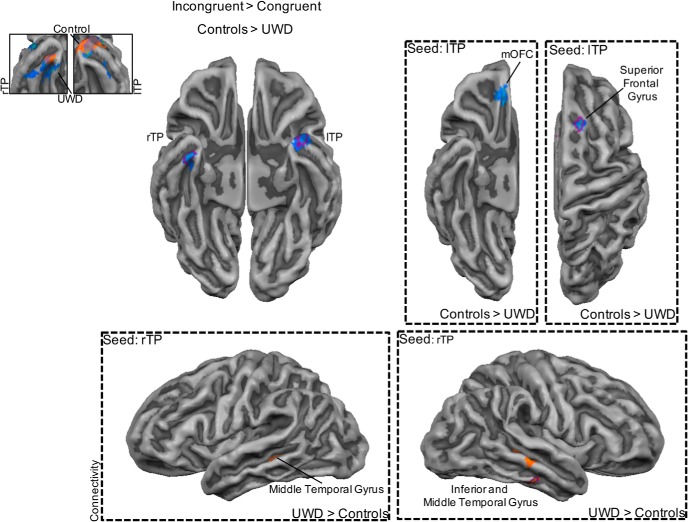
Disruption of the TP in the processing of incongruent threatening face–body compounds after BLA damage. Activity in the TP was reduced for the UWD group during perception of incongruent threatening face-body compounds (top left). Inset shows decreased bilateral TP activation for incongruent versus congruent face-body compounds in individuals with UWD, and increased bilateral TP activation for the same contrast in controls. Consistent with the dissociation between the frontal and temporal network, decreased functional connectivity was observed in individuals with UWD between the left TP and mOFC and superior frontal gyrus. The right TP showed increased coupling with the inferior temporal gyrus and bilateral middle temporal gyrus (right and bottom). Maps are cluster-size corrected except for the within-group maps that are shown with a threshold of *p* < 0.05 uncorrected for illustration purposes. Purple outline indicates that the cluster survived whole-brain cluster-size correction with an initial single voxel threshold of *p* < 0.005.

Next we investigated between-group differences in brain regions that showed differential activation for fearful versus happy bodies. Individuals with UWD compared with controls showed less activation in the left fusiform gyrus (BA 19) but more activation for fearful versus happy bodies in the right anterior part of the inferior parietal lobule (IPL; BA 40). Directly comparing incongruent with congruent face–body compounds revealed that individuals with UWD compared with controls showed more activation in the medial orbitofrontal cortex (mOFC; BA 11), ventromedial prefrontal cortex (vMPFC; BA 10), and dorsal medial prefrontal cortex (dMPFC; BA 9). However, individuals with UWD compared with controls showed less activation in the left (BA 38) and right (BA 21) TP. No significant between-group differences were found when directly contrasting task-irrelevant fear bodies versus task-irrelevant happy bodies. The results are presented in Figs. 2–4 and Table 7.

**Table 7.** Outcome of main between-group functional activation analyses

		Talairach coordinates						
	Hemisphere	*x*	*y*	*z*	Brodmann	*t*	*p*	Number of vertices
Fearful versus happy bodies regardless of the facial information								
UWD > controls								
Anterior inferior parietal lobule	RH	54	–29	32	40	4.606	0.000343	93
Controls > UWD								
Fusiform gyrus	LH	–41	–69	–12	19	–4.731	0.000268	33
Incongruent versus congruent face body compounds								
UWD > Controls								
Medial orbitofrontal cortex	RH	14	45	–12	11	4.724	0.000271	52
Ventromedial prefrontal cortex	RH	9	56	10	10	4.474	0.000446	51
Dorsal medial prefrontal cortex	RH	10	38	29	9	4.641	0.000320	42
Controls > UWD								
Temporal pole	RH	40	–4	–31	21	–4.486	0.000435	77
Temporal pole	LH	–33	6	–20	38	–4.430	0.000487	110

All clusters survive cluster-size correction except the anterior inferior parietal lobule and fusiform gyrus.

We ran an alternative analysis that focused solely on subcortical activation after BLA damage. To allow a fine-grained analysis, we ran the same contrasts as in the main analyses but masked the subcortical areas. No significant clusters emerged with or without spatial smoothing (4-mm Gaussian kernel).

### Functional connectivity

In a first analysis, we identified regions that showed functional connectivity with the IPL and the fusiform gyrus during the processing of fearful versus happy body regardless of the facial information. This revealed increased functional connectivity between the IPL and the subgenual anterior cingulate cortex (ACC; BA 24) in individuals with UWD compared with controls. Increased coupling between the fusiform gyrus and the anterior IPL (BA 40) was observed in individuals with UWD compared with controls, highlighting the importance of the latter region in threat processing.

Next, we established regions that showed functional connectivity with the mOFC, vMPFC, dMPFC, and left and right TP during the processing of incongruent versus congruent face–body compounds. Interestingly, individuals with UWD compared with controls showed decreased coupling between the mOFC and the posterior IPL (BA 7). Increased functional connectivity between the cuneus (BA 19), as well as the precuneus (BA 31), with the vMPFC was observed in individuals with UWD compared with controls. With the dMPFC as seed region, individuals with UWD compared with controls showed increased coupling with the vMPFC (BA 10) but decreased coupling with the superior temporal gyrus (BA 22) and TP (BA 38). Last, individuals with UWD compared with controls showed increased functional connectivity between the right TP and the inferior temporal gyrus (BA 20) and bilateral middle temporal gyrus (BA 21 and 22), and decreased functional connectivity between the left TP and mOFC (BA 11) and superior frontal gyrus (BA 6). Figs. 2–4 and Table 8 report the results from the functional connectivity analyses.

**Table 8.** Outcome of between-group functional connectivity analyses

		Talairach coordinates						
	Hemisphere	*x*	*y*	*z*	Brodmann	*t*	*p*	Number of vertices
Fearful versus happy bodies regardless of the facial information								
Seed: Inferior parietal lobule								
UWD > controls								
Subgenual anterior cingulate	RH	8	35	1	24	4.974	0.000167	50
Seed: Fusiform gyrus								
UWD > controls								
Anterior inferior parietal lobule*	LH	–54	–43	25	40	4.926	0.000183	51
Incongruent versus congruent face body compounds								
Seed: Medial orbitofrontal cortex								
Controls > UWD								
Posterior inferior parietal lobule	RH	40	–61	42	7	–4.648	0.000316	58
Seed: Ventromedial prefrontal cortex								
UWD > controls								
Precuneus	RH	7	–69	23	31	5.646	0.000047	21
Cuneus	RH	8	–82	26	19	4.650	0.000314	22
Seed: Dorsal medial prefrontal cortex								
UWD > controls								
Ventromedial prefrontal cortex	LH	–6	52	12	10	5.509	0.000060	29
Controls > UWD								
Superior temporal gyrus	LH	–47	20	3	22	–5.986	0.000025	108
Temporal pole	LH	–40	8	–25	38	–4.486	0.000435	43
Seed: Right temporal pole								
UWD > controls								
Inferior temporal gyrus	RH	55	–22	–17	20	5.564	0.000054	55
Middle temporal gyrus	RH	60	–25	–2	21	4.654	0.000312	88
Middle temporal gyrus	LH	–54	–36	–1	22	4.076	0.000994	37
Seed: Left temporal pole								
Controls > UWD								
Medial orbitofrontal cortex	RH	11	41	–12	11	–5.356	0.000080	36
Superior frontal gyrus	RH	19	26	52	6	–5.475	0.000064	38

* Did not survive cluster-size correction.

## Discussion

We investigated the effects of BLA damage on activity in the frontal and temporal networks during irrelevant threat processing. Results showed that BLA damage resulted in a differential impact on the BLA-frontal network and BLA-temporal network. In the BLA-damaged group compared with the control group, activity was increased for incongruent threatening face–body compounds in frontal midline regions (mOFC, vMPFC, dMPFC) but decreased in the bilateral TP. Functional connectivity analyses provided further indication of this differential effect and showed reduced coupling between frontal and temporal regions after BLA damage. Reduced coupling between the dMPFC and TP and superior temporal gyrus during the perception of incongruent threatening face–body compounds was observed in individuals with BLA damage compared with controls. Under similar conditions, we also observed decreased functional connectivity after BLA damage between the left TP and mOFC and superior frontal gyrus. In addition to the impact on frontal and temporal networks, results showed changes in IPL activity after BLA damage. We observed that activation for fearful versus happy bodily expression was increased in the IPL but decreased in the fusiform gyrus in BLA-damaged individuals compared with control individuals. Importantly, the IPL showed increased coupling with the subgenual ACC, whereas the fusiform gyrus showed increased functional connectivity with the IPL in the BLA-damaged group compared with the control group. Taken together, our results reveal the impact of BLA damage on a PFC-TP-IPL network during the processing of threat. This proposed PFC-TP-IPL network may be involved in several important processes that regulate confrontations with threat along three different axes, from ambiguity resolution to safety signaling and emotion regulation to the selection and execution of actions. Damage to the BLA could result in anomalous activity in all three nodes of the network and explain the previously observed hypersensitivity to threat (Terburg et al., 2012; de Gelder et al., 2014). We now discuss these effects and the influence of BLA damage in more detail.

### Temporal pole

Our results are consistent with existing knowledge on afferent and efferent connections and the functional role of the TP, a polymodal association area and part of the extended limbic system (Olson et al., 2007). Connections between TP and the nearby BLA have been reported in monkeys (Aggleton et al., 1980; Ghashghaei and Barbas, 2002), and similar connections were recently demonstrated in humans using *in vivo* probabilistic tractography (Bach et al., 2011) and meta-analytic connectivity modeling (Bzdok et al., 2013). The TP is also densely connected to midline regions, e.g., orbitofrontal cortex (Kondo et al., 2003), and the ventral, visual part of the TP receives input from extrastriate visual areas, e.g., inferior temporal regions (Markowitsch et al., 1985).

In view of findings showing that the TP is activated in a variety of social emotional tasks, from face perception to theory of mind, a recent review proposed a unifying role that could underlie the variety of results (Olson et al., 2007). The authors suggested that the TP binds valence to incoming visual signals, thereby providing the affective meaning to the percept. If so, one would expect that TP also drives the emotional labeling of possible ambiguous social cues. Indeed, increased TP activity was observed when participants view unique stimuli (Asari et al., 2008) or when participants labeled the emotion of two subtly different social interactions (Sinke et al., 2010). Importantly, this proposed perception–emotion linkage is similar to the role of the BLA in emotional coloring of a signal (Benarroch, 2015).

The TP together with the BLA might orchestrate the coupling between emotion and perception. This BLA-TP network establishes the emotional label and biases ongoing neural processes. The decreased activation to incongruent threatening face–body compounds, i.e., ambiguous threat, in the TP and decreased coupling with the mOFC after BLA damage could potentially underlie incorrect labeling of the compound as threat and subsequently bias upstream neural activity (e.g., midline PFC). This refers to a potential perceptual bias effect in which a task-irrelevant stimulus influences the percept of the task-relevant stimulus in the direction of the former (de Gelder and Bertelson, 2003). This effect is enhanced after BLA damage (de Gelder et al., 2014) and could thus be related to dysfunctional TP functioning and reduced cross-talk between temporal and frontal regions leading to impaired integration of perceptual and emotional processes.

### Prefrontal midline

The orbital and medial parts of the prefrontal midline that showed increased activation in the BLA-damaged group during incongruent or ambiguous threat are strongly connected to the BLA (Barbas, 2015) and have consistently been implicated in social-emotional processes (Likhtik and Paz, 2015). However, the different parts of the prefrontal midline have different connectivity patterns with regions within the AMG and have distinct but related roles (Barbas et al., 2003; Ghashghaei et al., 2007). Different functional consequences can emerge based on the precise location of the disruption in these amygdalae-prefrontal pathways (Myers-Schulz and Koenigs, 2012; Grupe and Nitschke, 2013). A disruption in the BLA-orbitofrontal pathway can lead to increased threat attention and hypervigilance (van Honk et al., 2016). On the other hand, disruption in the inhibitory control of the vMPFC on the BLA is thought to result in impaired safety learning (Grupe and Nitschke, 2013), consistent with the role of the MPFC-BLA pathway in safety signaling (Likhtik and Paz, 2015). This would hold especially for the ventral part of the MPFC, as the dorsal part has been associated with threat anticipation (Grupe and Nitschke, 2013; Klumpers et al., 2015a). For instance, when participants are confronted with a real-life threat and overcame their fear, vMPFC activation increased and was positively related to subjective fear (Nili et al., 2010). As the basolateral nuclei are central to these prefrontal pathways, damage to the BLA could lead to both hypervigilance to threat (Terburg et al., 2012) and impairment in safety signaling by increased attention to irrelevant threat (de Gelder et al., 2014).

Most often, threat signals are congruent and unambiguous, but sometimes the relevance and the actual threat significance of one cue conflicts with that of another and/or the interpretation of the context. The importance of the AMG, in particular the BLA, and the MPFC in these processes has been reported (Kim et al., 2003, 2004; Etkin et al., 2004, 2006; Brand et al., 2007; Neta et al., 2013; Nohlen et al., 2014). For example, the BLA code the subjective interpretation of the emotion of the face (Wang et al., 2014). Interestingly, when participants are interpreting ambiguous emotional faces, MPFC and BLA activation are inversely correlated (Kim et al., 2003). Similar findings of distraction by irrelevant threat (de Gelder et al., 2014) and increased reactivity to negative social emotional signals found after BLA damage (Terburg et al., 2012) have been obtained in individuals with mood and anxiety disorders (Mathews and MacLeod, 1994). Related to this, changes in connectivity of the MPFC with (parts of) the AMG have been found after early life stress (Malter Cohen et al., 2013), trauma (Thomason et al., 2015), and general anxiety disorder (Greenberg et al., 2013; Roy et al., 2013). Deficits in threat discrimination have been related to less differential responses in the vMPFC (Greenberg et al., 2013) and decreased MPFC-AMG connectivity (Cha et al., 2014). The absence of BLA input to the MPFC may lead to dysfunctional threat signaling and threat regulation.

### Inferior parietal lobule

Increased activation in the IPL for fearful bodily expressions regardless of the facial information was found after BLA damage. Moreover, under the same task conditions, increased coupling between the fusiform gyrus and IPL was observed in the BLA-damaged compared with the control group. The IPL has been implicated in action observation and representation (Rizzolatti and Matelli, 2003), maintaining attention (Malhotra et al., 2009), and fear processing (de Gelder et al., 2004; Sinke et al., 2010; Becker et al., 2012; Engelen et al., 2015). Several observations in the literature point to a possible link between the IPL and the representation and preparation of action during threat and the influence of the AMG on these processes. The right IPL has been implicated in responding to salient information in the environment (Singh-Curry and Husain, 2009). Directly influencing IPL activity during emotion body perception using online transcranial magnetic stimulation resulted in increased sensitivity for fearful bodily expressions (Engelen et al., 2015). A study that investigated face processing in two patients with complete bilateral AMG damage showed that the one patient that had both intact recognition of fearful facial expressions and startle responses to negative pictures also had increased activation in the premotor cortex and the IPL to fearful faces (Becker et al., 2012). In a recent study with the same population as in the present study, a ventral-to-dorsal processing shift during contextualized threat perception was observed after BLA damage (Hortensius et al., 2016b). Increased activation was observed in the anterior part of the IPL and other regions in the dorsal stream during the perception of neutral faces in a threatening scene. In the presence of BLA damage, a dorsal route instead of a ventral route might dominate the processing of task-irrelevant threat, probing reflexive reactions to threat (de Gelder et al., 2012). However, the IPL is a heterogeneous region and encompasses as many as five different clusters (Mars et al., 2011), each with distinctive roles (for example, Kwok and Macaluso, 2015). In the present study, both the anterior and posterior IPL were implicated in the neural circuitry after BLA damage, but under different task conditions and in different hemispheres. The anterior region is connected to premotor cortex and could serve as a crucial hub in the transition from perception to action. In contrast, the posterior part of the IPL is connected to the parahippocampal gyrus and activated during memory tasks. Which exact roles these different regions fulfill during threat perception and how these functional profiles change after BLA damage is unknown.

## Conclusion

To conclude, our study is the first to show the significance of a PFC-TP-IPL network in the functional integration of and reaction to threatening social stimuli by using a unique sample of individuals with BLA damage. Rather than attributing a function to the amygdalae as a whole, we clarify the specific contribution of one of its major nuclei in automatic action preparation in the IPL, dysfunctional emotion regulation processes in the prefrontal cortex, particularly the vMPFC, and less efficient ambiguity resolution in the TP.

## Synthesis

Reviewing Editor: Carmen Sandi, Swiss Federal Institute of Technology Decisions are customarily a result of the Reviewing Editor and the peer reviewers coming together and discussing their recommendations until a consensus is reached. When revisions are invited, a fact-based synthesis statement explaining their decision and outlining what is needed to prepare a revision will be listed below. The following reviewer(s) agreed to reveal their identity: Helen Barbas.

The current manuscript aims to determine the role of the basolateral amygdala (BLA) in social threat processing. It includes functional imaging results in 5 patients with a rare disease (Urbach-Wiethe Disease) that causes calcification lesions in the brain, in this case quite selective BLA damage. These and control subjects were studies while engaged in a task designed to assess automatic perception of threat in a frontal/temporal network that also involves the amygdala. Main findings include reduced response to stimuli in the temporal pole and increased response in the medial prefrontal and medial orbitofrontal cortex. The authors have interpreted this lowered activity in temporal regions plus increased activity in frontal regions to indicate an imbalance of the threat perception factors ambiguity resolution, safety signaling, and action.

The reviewers and editors find the study interesting and relevant. However, we consider that there are a few issues that need clarification:

1. Details on the structure of the task need to be clarified. The authors write that a fixation cross could change into a red circle in order to counteract motor response in other trials. It is not clear which trials the authors mean, as the task is not very precisely described. It is not clear how the authors are using the visual stimulus to counteract the motor response. It would help if the authors clean up this section and describe the task in greater detail.

2. Please, clarify why you chose not to include a neutral condition, and instead contrast fearful and happy emotions. This is very important given that the contrast was done on between a social, emotional stimulus (emotional face/body) and a nonsocial stimulus (gray shape over the face/body), meaning that the final contrast between target stimulus and control is in effect “social/ emotional stimulus > nonsocial/nonemotional”. The discussion should deal with this issues as, should the authors have included a neutral emotional stimulus, the contrast could have been “social /emotional > social/nonemotional” and allowed to isolate the effect of emotion.

3. Lines 222-226: clarify the design for analysis for a general audience by avoiding excessive statistical jargon.

4. Throughout the discussion there are statements that do not include references in support of the arguments: e.g., Lines 345-6, 'Resolving such affective ambiguity is sustained by the TP'; the same goes for the next sentence (lines 346-348). This is particularly important since the idea of 'resolving ambiguity' is illustrated in figure 5 and the legend without references in the text. An update of the literature of the circuits through the amygdala/thalamus/cortex in primates (not only in rodents) would also improve the discussion.

5. The article indicates that the authors decided on a liberal initial single voxel threshold of p = 0.01. A reference that argues against using this threshold (Woo et al 2014, cited on page 12 here) is included. It would be important, on the contrary, that a reference is provided on support that this more liberal threshold can provide the same level of robust findings.

Minor issues:

1. There are several grammatical errors (e.g. page 5 line 96, correspondents should be corresponds; page 6 line 119-121, the sentence seems to be incomplete), and the manuscript could be improved greatly with a careful read-through.

2. Numbers greater than 10 are sometimes written out as words, sometimes listed as digits.

3. UWD is listed as an abbreviation for Urbach-Wiethe disease, and refer to patients with UWD as UWDs. We would suggest writing “patients (or individuals) with UWD” instead of using the abbreviation of the disease itself to preserve patients' dignity. In the figures, we would suggest using the label “UWD > Controls”.

4. Line 131, 'The BLA has an inhibitory influence on the MPFC..', it is important to clarify that this is most likely due to activation of inhibitory neurons in MPFC. There is also evidence in humans that pathways from the MPFC exercises inhibitory control over the amygdala. How do the authors interpret this information in the context of their findings?

5. Throughout Results the authors refer to the cortex in general terms (e.g., mOFC, vMPFC, dMPFC and TP). It would be helpful to say which areas are included in each of these regions.

6. Line 363, 'Our findings indicate...' is too strong; it is more appropriate to say: Our findings suggest.

7. Figure 1: It is necessary to add a key to indicate which nucleus is represented by each color in the reconstructed amygdala maps; also, insert indicator lines to identify the axes (dorsal/ventral/medial/lateral), and add a scale.

8. Intro, line 96, change correspondents to: corresponds

9. Lines 119-121, is this a question?

10. Line 281, '..and interior temporal..' should probably say inferior, or anterior, depending on which applies.

11. Line 321-322: , should be Figures 2-4... and report.

12. Please correct several other typographic errors throughout the ms.
